# A modified sentinel node and occult lesion localization (SNOLL) technique in non-palpable breast cancer: a pilot study

**DOI:** 10.1186/s13046-015-0230-x

**Published:** 2015-10-06

**Authors:** Giulia Anna Follacchio, Francesco Monteleone, Paolo Anibaldi, Giuseppe De Vincentis, Silvia Iacobelli, Raffaele Merola, Valerio D’Orazi, Massimo Monti, Vittorio Pasta

**Affiliations:** Department of Radiological, Oncological and Anatomo-Pathological Sciences, Nuclear Medicine Unit, “Sapienza” University of Rome, Rome, Italy; Breast Unit, Department of General Surgery, “San Camillo de Lellis” Hospital, Rieti, Italy; Department of Surgical Sciences, “Sapienza” University of Rome, Rome, Italy; Department of General Microsurgery and Hand Surgery, “Fabia Mater” Hospital, Via Olevano Romano 25, 00171 Rome, Italy

**Keywords:** Non-palpable breast cancer, sentinel lymph node, SNOLL, radioguided surgery, imaging probe

## Abstract

**Background:**

The spread of mammographic screening programs has allowed an increasing amount of early breast cancer diagnosis. A modern approach to non-palpable breast lesions requires an accurate intraoperative localization, in order to achieve a complete surgical resection. In addiction, the assessment of lymph node status is mandatory as it represents a major prognostic factor in these patients. The aim of this study is to evaluate the reliability of a modified technical approach using a single nanocolloidal radiotracer to localize both sentinel node and breast occult lesion.

**Methods:**

Twenty-five patients with a single non-palpable breast lesions and clinically negative axilla were enrolled. In the same day of surgery, patients underwent intratumoral and peritumoral administration of ^99m^Tc-labeled nanocolloid tracer under sonographic guidance. A lymphoscintigraphy was performed to localize the sentinel lymph node and its cutaneous projection was marked on the skin in order to guide the surgeon to an optimal incision. During surgery an hand-held gamma-detection probe was used to select the best surgical access route and to guide localization of both occult breast lesion and sentinel lymph node. After specimen excision, the surgical field was checked with the gamma-probe to verify the absence of residual sources of significant radioactivity, thereby ensuring a radical treatment in a single surgical session and minimizing normal tissue excision.

**Results:**

Both targeted breast lesion and sentinel lymph node were localized and removed at the first attempt in every patients and histopathological diagnosis of malignancy was confirmed in 25/26 samples. Non-palpable lesions were included within the surgical margins in all patients and in all samples surgical margins were free from neoplastic infiltration thus avoiding any further reintervention. Only two patients showed metastatic involvement of sentinel lymph node.

**Conclusions:**

The modified sentinel node and occult lesion localization (SNOLL) technique performed with a single injection of nanocolloidal radiotracer has shown an excellent intraoperative identification rate of both non-palpable lesion and sentinel lymph node. This procedure offers, as opposed to standard techniques, an accurate, simple and reliable approach to the management of non-palpable breast cancer.

## Background

Over the last decades, the frequency of non-palpable breast tumors has increased due to widespread of mammographic screening programs and the development of more accurate imaging technique [1–3]. As a result of this trend, it has been reported a decrease in the mean size of neoplastic lesions and a reduction in axillary lymphatic involvement [4, 5]. The constant increase of early, non-palpable breast cancer diagnosis requires a comprehensive approach in order to achieve optimal surgical treatment, as we already highlighted in breast sarcomas [6, 7]. An ideal intraoperative localization procedure of non-palpable breast lesions should enable the surgeon to accomplish a complete excision in a single surgical session while avoiding excessive removal of normal tissue. Since its development**,** radioguided occult lesion localization (ROLL) technique has been evaluated as a more reliable procedure to guide the surgical resection of non-palpable breast lesions than hooked wire technique [8–13]. In patients with early breast cancer and non-palpable lesion the assessment of axillary lymph node status is one of the major prognostic factor [14]. In order to assess axillary lymph node status, sentinel limph node (SLN) biopsy is now a widely accepted procedure for staging patients with early breast cancer, thus avoiding them unnecessary complete axillary dissection [15–19]. The possibility to perform ROLL and SLN biopsy in the same surgical session, a procedure known as sentinel node and occult lesion localization (SNOLL) technique, has been evaluated in several studies by using different tracers and sites of injection [20–24]. The aim of this study is to evaluate the reliability of a modified SNOLL approach characterized by a single injection of unique nanocolloidal radiotracer for simultaneous occult breast lesion and sentinel lymph node localization.

## Materials and methods

### Patients

Between November 2012 and October 2014, 25 female patients with a single non-palpable breast lesion, were enrolled for this study, based on preoperative cytologic or histologic evidence. None of the patients had clinical evidence of axillary disease, and all underwent radioguided conservative surgery at the Departments of Radiological Sciences and Surgical Sciences at “Sapienza” University of Rome, Italy. The protocol was approved by the institutional review committee, informed consent was obtained from the patients and all procedures were in accordance with the ethical standards of the responsible institutional committee on human experimentation and with the Helsinki Declaration of 1975, as revised in 1983.

Instrumental diagnosis was based on X-ray mammography (X-RM) and breast ultrasound (US); in selected cases a breast magnetic resonance imaging (MRI) was performed. Images were interpreted in accordance with the breast imaging reporting and data system (BIRADS) lexicon by radiologists experienced in breast imaging. Patients with non-palpable breast lesion classified as BIRADS 4, BIRADS 5, BIRADS 6 were included in the study; preoperative diagnosis of malignancy or suspect malignancy was obtained by Fine Needle Aspiration Cytology (FNAC) or Core Biopsy. Patients with diffuse microcalcifications, multifocal or multicentric lesions, previous breast excisional biopsy or radiotherapy were excluded.

### Modified sentinel node and occult lesion localization (SNOLL) technique

**Fig. 1:**
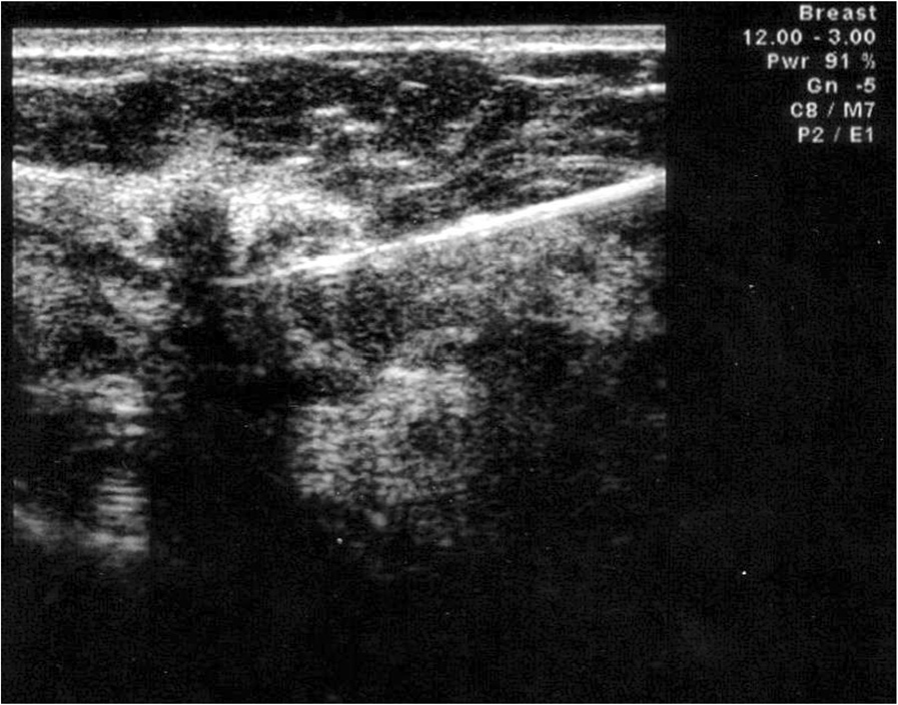
US-guided intralesional injection of nanocolloidal radiotracer

**Fig. 2:**
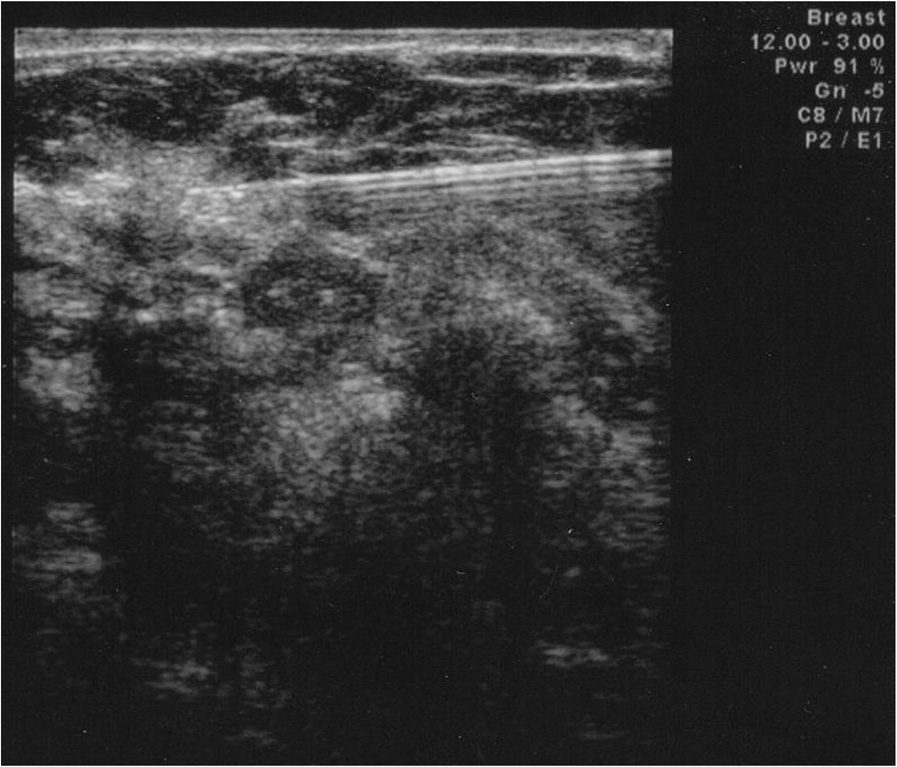
US-guided perilesional injection of nanocolloidal radiotracer

The modified SNOLL procedure was performed in a one-day protocol with scheduled surgery. We used a single nanocolloidal tracer (Nanocoll®, Gipharma srl, Saluggia-VC-, Italy, average particle size <80 nm) labeled with ^99m^Technetium (^99m^Tc); average radioactivity dose was 35 MBq (range 20–70 MBq) in a volume of 0.4 ml. On the same day of surgery, the nanocolloidal radiotracer was administered by the nuclear physician under US guidance provided by a breast radiologist with 20 years of experience in the field of breast imaging (10.5 MHz linear probe, Aplio XV, Toshiba); through a 22 G needle, half the dose of tracer was injected intratumorally (Fig. 1) in order to mark the clinically occult breast lesion, while the other half was equally divided between the opposite poles of the lesion (peritumoral injection) to identify the lymphatic drainage pathway towards SLN (Fig. 2). The entire procedure was carried out without extracting the needle between the two administrations. After radiotracer injection, all patients were sent to the Nuclear Medicine Unit where lymphoscintigraphy was performed: images were acquired with the patient positioned supine with the arm ipsilateral to the lesion in maximum abduction, using a dual-head gamma camera (Millenium MG, GE Medical Systems, Wisconsin, USA) with low-energy, high-resolution parallel-hole collimators. Early dynamic acquisitions were obtained in oblique anterior (OA) projection for 20 minutes (64×64 matrix, 1 image/minute), then static images were performed in OA and lateral position with an acquisition time of 300 seconds in a 128×128 matrix. After the visualization of the lymphatic drainage pathway and SLN, its cutaneous projection was identified thanks to ^57^Co scintigraphic tracer and marked on the skin with permanent ink to provide a guide for an optimal surgical incision.

### Surgery

**Fig. 3:**
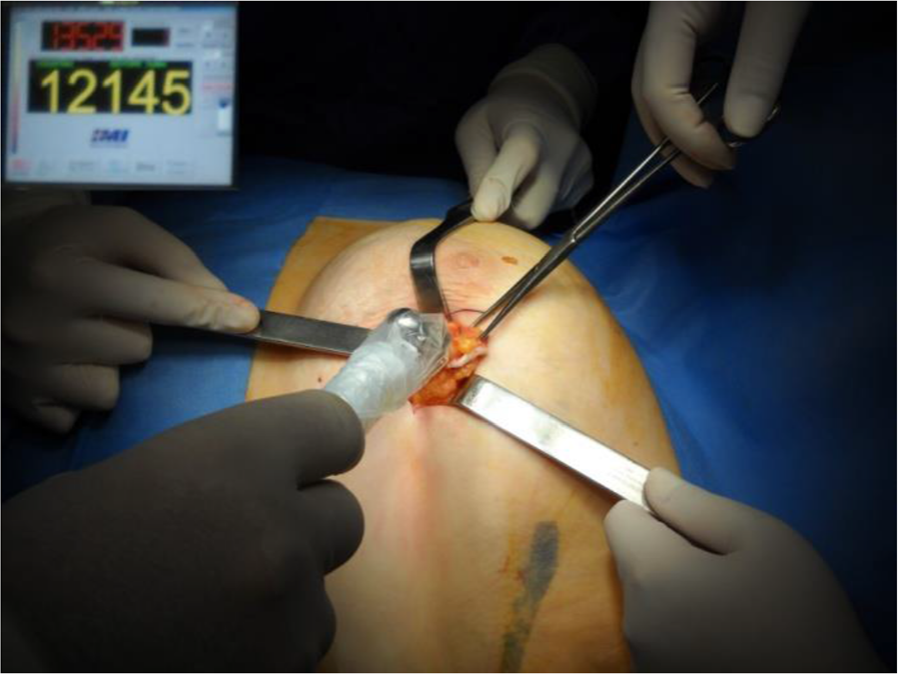
Intraoperative radio-guided excision of occult breast lesion

**Fig. 4:**
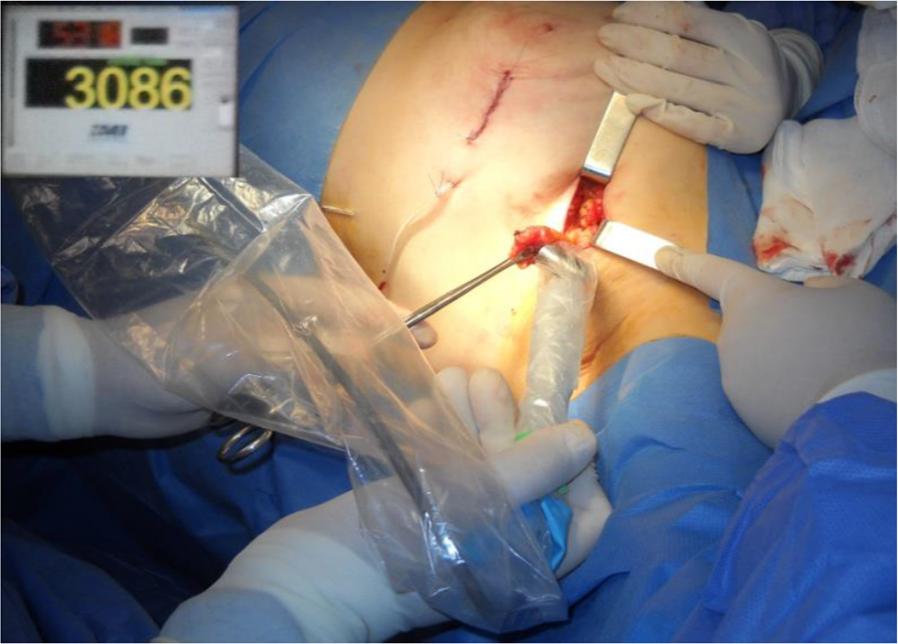
Intraoperative radio-guided localization and excision of sentinel node

Each patient underwent conservative surgery on the same day of radiotracer injection. A hand-held gamma-detection probe (GDP - Node Seeker™ 800, IntraMedical Imaging, CA, USA) set to ^99m^Tc photo-emittance peak (140 keV ± 20 %) was used by the surgeon on intact skin to select the best surgical access route, then the same probe was introduced in the incision at different angles to detect the area of maximal radioactivity in the surgical field and to define margins of resection (Fig. 3). After radio-guided excision, breast tissue specimen was oriented on three points and double-checked with GDP once moved away from the surgical field, then γ-probe was used to verify the absence of residual areas of significant radioactivity in resection field; the surgeon applied the same research technique to identify and remove SLN (Fig. 4). If the breast lesion was in the upper outer quadrant, the incision used to remove the lesion was the same to access also the SLN, thus achieving a better cosmetic outcome; if the lesion was in another quadrant, a separate axillary incision was performed to remove SLN.

### Pathological examination

Histological examination of the surgical specimen was carried out by a pathologist specialized in breast diseases. Breast tissue specimens were examined macroscopically to determine dimensions of the excised tissue, presence and size of the lesion and microscopically to assess the nature of the lesion, resection margins’ size and their eventual neoplastic infiltration. SLN was analyzed in serial sections with haematoxylin-eosin staining and immunohistochemistry with anti-cytokeratine antibodies (clone MNF116) to assess the presence of macrometastases, micrometastases or isolated tumor cells.

## Results

Initially, fourty patients with a clinically occult breast lesion detected by X-RM or US imaging and charachterized by FNAC or Core Biopsy were consecutively evaluated; all patients had no clinical evidence of axillary disease. According to trial exclusion criteria, 6 patients were excluded because of diffuse microcalcifications at X-RM, 3 patients were excluded because multifocal or multicentric lesions were detected during MRI evaluation, 2 patients were excluded for previous excisional biopsy on the same breast, 1 patient was excluded for previous breast radiotherapic treatment and 3 patients were excluded because non-malignant lesion was diagnosed by FNAC or Core Biopsy, for a total of 15 patients excluded. The remaining 25 patients with imaging report (X-RM, US and MRI) of single non-palpable breast lesion and clinically negative axilla were enrolled for the study. One patient showed a clinically occult breast lesion in both breasts so that she underwent bilateral SNOLL procedure. As a result, we targeted a total of 26 clinically occult breast lesions. Mean age of patients was 60 years (range 43–81 years); 5 patients (20 %) were in menopause and 20 patients (80 %) were postmenopausal. Preoperative lesion characteristics are shown in Table 1.

**Table 1:** Preoperative lesion’s characteristics

		No. of lesions	%
	Age		
Mean	60		
Range	43-81		
Breast quadrant			
Upper outer		17	65 %
Inferior inner		2	7 %
Upper inner		6	24 %
Inferior outer		1	4 %
Radiological diagnosis			
BI-RADS 4		13	50 %
BI-RADS 5		11	42 %
BI-RADS 6		2	8 %
Cytological (C) or histological (B) diagnosis			
C3		4	15 %
C4		6	23 %
C5		8	31 %
B4		2	8 %
B5		6	23 %

After intratumoral radiotracer injection, clinically occult breast lesion was successfully identified by using the γ-detection probe and removed at the first surgical session in every patient. The evaluation of surgical field by using γ-probe demonstrated the absence of residual areas of significant radioactivity in resection field in all cases. Histological examination of the surgical specimens confirmed malignancy in 25 patients (96 %); in one patient there was evidence of non-malignant lesion (this patient had a breast lesion classified as BIRADS 4 at imaging and C3 at FNAC). Every breast lesion (mean size 8 mm, range 4–18 mm) was included within the surgical margins, which were free from neoplastic infiltration, thus avoiding any further re-intervention. The closest resection margin was 1 mm in 3 patients. Complete results of the final histological examination are shown in Table 2.

**Table 2:** Final histopathological findings

		No. of Patients	%
Dimensions			
Mean	8 mm		
Range	4-18 mm		
Histological diagnosis			
Invasive ductal carcinoma (IDC)		6	23
IDC + ductal carcinoma (DC) *in situ*		11	42
DC *in situ*		4	15
Invasive lobular carcinoma (ILC)		2	7
Tubular Carcinoma		1	4
Tubular Carcinoma +		1	4
DC *in situ*			
No evidence of malignancy		1	4
Grading			
G1		9	36
G2		10	40
G3		6	24
Resection margins (mm)			
1		3	12
2		5	20
3		0	0
4		3	12
5		6	24
6		2	8
7		1	4
8		1	4
9		0	0
>10		4	16

Lymphoscintigraphy showed axillary drainage pattern in all patients; a single SLN was identified in 24 patients, while in two patients were localized two SLN. Lymphoscintigraphic findings were always confirmed during surgery by using γ-probe and SLN was found and removed in every patient.

A total of 35 lymph nodes were isolated at final histopathological examination (average excised lymph nodes per patient 1,4); 32/35 lymph nodes were free from metastatic involvement, two SLN showed macrometastases (>2 mm) so patients were addressed to complete axillary dissection, while in one patient SLN showed isolated tumor cells (Table 3) whose malignant potential and prognostic significance are still under debate [25–27]. Post-operative TNM classification is shown in Table 4. The entire modified SNOLL procedure was well tolerated by all patients and no complications were observed.

**Table 3:** Status of removed lymph-nodes (LNs)

	No. of LNs	%
Free from metastatic involvement	32	91
Isolated Tumor Cells	1	3
Micrometastases	0	0
Macrometastases	2	6

**Table 4:** Postoperative pTNM classification

	No. of patients	%
pT		
pTis	2	8.33
pT1a	3	12.5
pT1b	15	62.5
pT1c	3	12.5
pT2a	1	4.16
pN(sn)		
pN0	21	87.5
pN0 (i+)(sn)	1	4.16
pN1 mi(sn)	0	0
pN1a	2	8.33

## Discussion

Clinical presentation of breast cancer is changing thanks to mammographic screening programs and greater awareness among women. The increasing frequency of non-palpable breast lesions, characterized by low risk of metastatic lymph nodes at diagnosis [28], requires an approach aimed at both accurate intraoperative localization of occult neoplastic lesions and reliable SLN identification. As a matter of fact, several imaging techniques are used to detect metastasis from different types of cancers [29]. Gamma detection probe is more frequently indicated for two surgical procedures: SLN sampling (for breast cancer and melanomas) and minimally-invasive radioguided surgery of parathyroid adenomas [30, 31].

In this study, the modified SNOLL technique characterized by simultaneous intra- and peri-tumoral US-guided injection of nanocolloidal tracer has shown an excellent localization rate of both occult breast lesion and SLN.

The choice of a single-day protocol characterized by radiotracer injection and lymphoscintigraphy performed on the morning of surgery, when compared with approaches previously described such as the one proposed by Feggi et al. [20], allowed to improve time management and to reduce radioactive exposure to patients.

In addition, radiotracer administration under US guidance was easy to perform and provided a real-time control of needle position, permitting an accurate injection of the tracer. Nanocolloidal radiotracer injected intratumorally remains in the inoculation site for the necessary time to enable the surgeon to select the optimal surgical access route to the lesion and to perform an efficient tumor localization and excision through the constant orientation provided by γ-probe. Furthermore, after each radio-guided excision the surgeon was able to perform a check on the surgical field to confirm the absence of neoplastic tissue residual immediately. This approach allowed a limited breast conserving treatment and ensured disease-free resection margins in a single surgical session. In our series, as demonstrated by histopathological analyses of the surgical specimens, the modified SNOLL procedure permitted a complete excision of the lesion at the first attempt in every patient, with no need of re-intervention for involved resection margins. Limited breast tissue excision allowed a better cosmetic outcome for patients improving patient compliance and potentially reducing subsequent plastic surgery. SLN identification obtained with bipolar perilesional tracer injection, allowed an accurate study of lymphatic drainage pathway and a selective surgical removal of the SLN identified with γ-probe, avoiding unnecessary complete axillary dissection and its surgical complications, such as chronic arm lymphedema and paraesthesias [32–35]. In our series, 88.9 % of patients had no metastasis at the frozen section histological examination of the SLN and only in two patients complete axillary dissection was required because of SLN macrometastasis detection.

## Conclusions

In conclusion, this pilot study shows that, as opposed to standard techniques (i.e., radioguided sentinel lymph node biopsy performed on the day prior surgery and radio-guided occult lesion localization performed on the day of surgery), the modified SNOLL technique is an accurate and easy-to-perform procedure. Indeed, it allows, following intra- and peri-tumoral injection of a single nanocolloidal radiotracer under US guidance, both tumor excision and SLN removal in a single surgical session reducing re-intervention rate and unnecessary complete axillary dissection. It is worth to remember that the procedure doesn’t require any particular radioprotection measure due to the extremely low-dose exposition of the operating staff, as assessed by Cremonesi [36]. This procedure improves resource and time management of early, non-palpable breast cancers increasing comfort for patients.

## Abbreviations

SNOLL: sentinel node and occult lesion localization
ROLL: radioguided occult lesion localization
SLN: sentinel limph node
X-RM: X-ray mammography
US: ultrasound
MRI: magnetic resonance imaging
BI-RADS: breast imaging reporting and data system
FNAC: fine needle aspiration cytology
nm: nanometer
^99m^Tc: technetium-99 m
MBq: megabecquerel
MHz: megahertz
G: gauge
OA: oblique anterior
^57^Co: Cobalt-57
GDP: gamma-detection probe
keV: Kiloelectron-volts
